# Laccase-derived lignin compounds boost cellulose oxidative enzymes AA9

**DOI:** 10.1186/s13068-017-0985-8

**Published:** 2018-01-17

**Authors:** Lívia Brenelli, Fabio M. Squina, Claus Felby, David Cannella

**Affiliations:** 1 Faculty of Science, Department of Geosciences and Natural Resource Management, University of Copenhagen, Frederiksberg C, Denmark; 2 Brazilian Bioethanol Science and Technology Laboratory (CTBE), Brazilian Center for Research in Energy and Materials (CNPEM), Campinas, Sao Paulo, Brazil; 3 Programa de Processos Tecnológicos e Ambientais, Universidade de Sorocaba, Sorocaba, Brazil, Sorocaba, Brazil; 4 Present Address: Interfaculty School of Bioengineering, Université Libre de Bruxelles (ULB), Campus Plaine CP242, Boulevard du Triomphe, Brussels, 1050 Belgium

**Keywords:** Laccase, LPMO, Sugarcane bagasse, Wheat straw, Lignin, Cellulose oxidation

## Abstract

**Background:**

The discovery of lignin as activator for the redox enzyme lytic polysaccharide monooxygenases (LPMOs) for the oxidation of cell-wall polysaccharides opens a new scenario for investigation of the interplay between different lignocellulose-degrading enzymes. The lignin-active enzymes in one hand, and the carbohydrate active in the other, are linked through a variety of electrons carrier molecules either derived from lignin or enzymatically transferred. Likewise, in nature, many lignocellulose-degrading organisms are expressing those enzymes simultaneously, and we wanted to test if a major commercial available lignin oxidase enzyme, i.e., laccase could benefit and synergize the activity of the LPMOs by depolymerizing the insoluble lignin.

**Results:**

In this work, two fungal laccases together with a mediator (ABTS) were used to isolate low-molecular-weight lignin from lignocellulosic biomass. The isolated lignins were used as electron donors for activation of LPMOs. A direct correlation between the low-molecular-weight lignin isolated with laccases and an increased activity of a cellulolytic cocktail containing LPMO was found when pure cellulose was hydrolyzed. We then tried to implement existing commercial cellulases cocktail with laccase enzymes, but under the conditions tested, the co-incubation of laccases with LPMOs showed a substrate competition towards oxygen inhibiting the LPMO. In addition, we found that laccase treatment may cause other modifications to pure cellulose, rendering the material more recalcitrant for enzymatic saccharification.

**Conclusions:**

Laccase-mediated system was able to depolymerize lignin from pre-treated and native sugarcane bagasse and wheat straw, and the released phenolic molecules were able to donate electrons to LPMO enzymes boosting the overall enzymatic hydrolysis of cellulose. Likewise, other poly-phenol oxidase, we might have just started showing possible pros or cons in applying several oxidase enzymes for a simultaneous degradation of cellulose and lignin, and we found that the competition towards oxygen and their different consumption rates must be taken into account for any possible co-application.

**Electronic supplementary material:**

The online version of this article (10.1186/s13068-017-0985-8) contains supplementary material, which is available to authorized users.

## Background

Over the last century, our dependence on non-renewable fossil-based fuels has led to concerns over climate change effects, and increased the interest of creating a sustainable bioeconomy. Carbon-based but fossil-free technologies such as the enzymatic conversion of lignocellulosic materials to fuels and value-added products are, therefore, gaining significant attention [1, 2]. Lignocellulose is a recalcitrant and complex material composed mainly of cellulose, hemicellulose, and lignin. The enzymatic conversion of this material has been intensively studied, dividing it into two major classes of reactions: oxidative modification/depolymerization of lignin and hydrolysis of polysaccharides. This paradigm was valid until the recent discovery of an oxidative enzyme active on polysaccharides, named lytic polysaccharide monooxygenase (LPMO). Today, LPMOs have been found widespread in the Tree of Life: from bacteria to fungi, and often together with lignin-active enzymes, e.g., ligninase, manganese, and versatile peroxidase and laccase, or in some organisms also with glucose–methanol–choline (GMC) oxidoreductases, e.g., cellobiose dehydrogenase (CDH), pyranose oxidase, poly-phenol oxidases, or galactose oxidase [3, 4]. LPMOs oxidize glucose-based oligosaccharides resulting in a non-reducing end and a C1-oxidized end, or a reducing end and an oxidation of the C4 at the non-reducing terminal [5, 6]. The products of the subsequent actions of exo-cellulases and β-glucosidases are monomeric glucose molecules and their oxidized forms: gluconic acid and gemdiol 4-ketoaldose (used as markers of LPMOs activity), from C1 and C4 oxidation, respectively [7]. New generations of commercial cellulolytic cocktails contain LPMO to exploit the oxidases/hydrolase synergism [8], which has a positive impact on the industrial ethanol production, and consequently benefiting the overall CO_2_ emission of the processes [9, 10]. LPMOs are copper-dependent enzymes active only in the presence of molecular oxygen [11], and an electron donor, which can be delivered in different forms: from enzymes (GMC oxidoreductases) [12, 13], from plant cell-wall-derived lignins or phenolic compounds [9, 14, 15], and recently from light-activated photosynthetic pigments [16, 17]. Moreover, another elucidated mechanism shows that lignin and low-molecular-weight lignin-derived compounds (LMWLDC) from plant cell wall can deliver electrons to LPMO [18]. In a long-range electron transfer, bulk lignin works as a source of electrons, while LMWLDC shuttles the electrons to LPMOs and being oxidized; after the oxidation, these LMWLDC are reduced back by an electron donation from the bulk lignin, thus capable to restart a new electron delivery [13, 14, 18]. An interesting aspect is that these LMWLDC, i.e., caffeic acid and sinapic acid, which deliver electrons to LPMO, might be obtained from the plant cell wall upon the activity of lignin-active enzymes or class II peroxidases (POD) systems [19], which are co-expressed in fungi together with LPMOs. So far, these enzymes have been individually studied and few reports have focused on the interplay or synergism between different oxidases upon lignocellulose degradation, and mainly investigating the electron transfer between CDH and LPMO, and more recently, the phenols mediated poly-phenol oxidase (PPO) electron transfer to LPMO [4]. In this context, many aspects still need to be investigated. Laccases are multicopper oxidases catalyzing the oxidation of phenolic compounds by a one-electron transfer, driven by the reduction of molecular dioxygen to water [20]. Its potential involvement in lignin depolymerization and/or modification in vivo has opened the field to its potential biotechnological applications [21]. In the presence of small molecules such as 2,2′-azino-bis (3-ethylbenzthiazoline-6-sulfonic acid) (ABTS), acting as electron shuttle, laccases are able to oxidize a wider range of substrates, and it is referred to as laccase–mediator systems (LMS) [22]. Studies employing different laccases and lignocellulose materials have confirmed the potential of LMS for lignin depolymerization and/or modification [23–25].

In this work, we combine the oxidation of lignin by fungal laccases with the cellulose oxidation done by LPMO and connect the two oxidative systems by means of low-molecular-weight lignin-derived compounds (LMWLDC) functioning as electron shuttles. Steam-exploded sugarcane bagasse (SCB) and hydrothermal-treated wheat straw (WS) were incubated with low or high redox laccase from *Myceliophthora thermophila* and *Trametes villosa* combined with ABTS as mediator to produce LMWLDC. Gel permeation chromatography (GPC) and RP-HPLC analytical instruments were used to monitor LMS assays. The liquid phase, rich in LMWLDC, was used to boost the activity of LPMO in a commercial cellulase cocktail. Furthermore, co-incubation of cellulase and laccase was performed to assess potential oxygen competition. A special gas sealed reactor equipped with an oxygen sensor was used for the enzymatic hydrolysis and oxidation of lignocellulose.

## Materials and methods

### Materials

Steam-exploded sugarcane bagasse (SCB) was kindly provided by The Engineering School of Lorena (EEL-USP) and details of the process, and the washing and pressing steps to remove solubilized sugars and degradation products were described previously [26]. Wheat straw was hydrothermally pre-treated (WS) in an oil bath at 194 ± 1.5  °C with a residence time of 20 min at 10% dry matter content in water using a Parr reactor with 100 mL capacity. A washing and pressing step was applied after the pre-treatment to remove solubilized sugars and degradation products. The SCB and WS were dried at 30 °C and ground in a coffee grinder for 2 min (particle size distribution ranging from 0.5 to 5 mm) prior to further use. The chemical composition determined using the NREL method for lignocellulose biomasses [27] was reported to be 52% glucan, 6% xylan, 24% lignin, 0.2% galactan, 0.5% arabinan for pre-treated SCB and 54% glucan, 5% xylan, and 32% lignin for pre-treated WS. The chemical composition of the raw WT and SCB was described previously in [14] and [26], respectively. Microcrystalline Avicel cellulose was used as model substrate for enzymatic hydrolysis and was purchased from Sigma-Aldrich Co. (MO, USA).

### Enzymes and chemicals

The two commercial laccases used in this study were supplied from Novozymes A/S (Bagsvaerd, Denmark): *Myceliophthora thermophila* laccase (MtL) with a low redox potential (450 ± 10 mV) [25] and *Trametes villosa* laccase (TvL) with a high redox potential (790 ± 10 mV) [28]. The laccase activities were measured by oxidation of 1 mM ABTS to its radical form by monitoring the absorbance at 420 nm (*ε* = 36 000 M^−1^ cm^−1^) in 0.5 M sodium acetate buffer pH 5.0 at 20 °C. One unit of activity was defined as the amount of enzyme required to oxidize 1 µmol of ABTS per minute. The cellulolytic commercial cocktail containing LPMO activity employed in this study was Cellic^®^ CTec2 (Novozymes A/S, Bagsvaerd, Denmark). The protein content of the enzymatic preparation was 160 mg protein g^−1^, as determined by the bicinchoninic acid (BCA) method. The cellulase activity was 120 FPU g^−1^ of preparation measured by the filter paper assay. Cellic^®^ CTec2 was stored at 4 °C. ABTS was purchased from Sigma-Aldrich Co. (MO, USA).

### Laccase-mediated system (LMS) treatments for production of LMWLDC

Enzymatic treatments with laccases were performed always in the presence of ABTS as mediator molecule if not otherwise stated, and referred to as laccase–mediator system treatments (LMS). LMS treatments were performed on SCB, WS, and Avicel at 5% (w/v) dry matter (DM) in 0.05 M sodium acetate buffer pH 5.0 in 20 mL reaction volume at 40 °C, 500 rpm shaking for 6 h. MtL or TvL laccases were added at a dosage of 10 U g cellulose^−1^ DM (corresponding to 500 µU mL^−1^ of enzyme) and the ABTS mediator was dosed at a final concentration of 1 mM. The incubations were conducted in the presence of an external air supply (flask unsealed). Incubations lacking laccase and containing only the ABTS mediator were used as controls. After the incubations, laccase activity was stopped by adding NaN_3_ at a final concentration of 0.05% (w/v), and oxygen consumption measurements using an oxygen electrode chamber (Oxy-Lab Hansatech^®^) confirmed that laccase enzymes were inactivated. The reactions were centrifuged (1200*g*, 10 min at 20 °C) and the supernatants rich in LMWLDC were separated and stored at 4 °C. All pellets containing the leftover biomass were washed three times with pure water to remove residual laccases and mediators and stored at 4 °C prior further enzymatic hydrolysis.

### Enzymatic hydrolysis of Avicel supplemented with LMWLDC

**Fig. 1:**
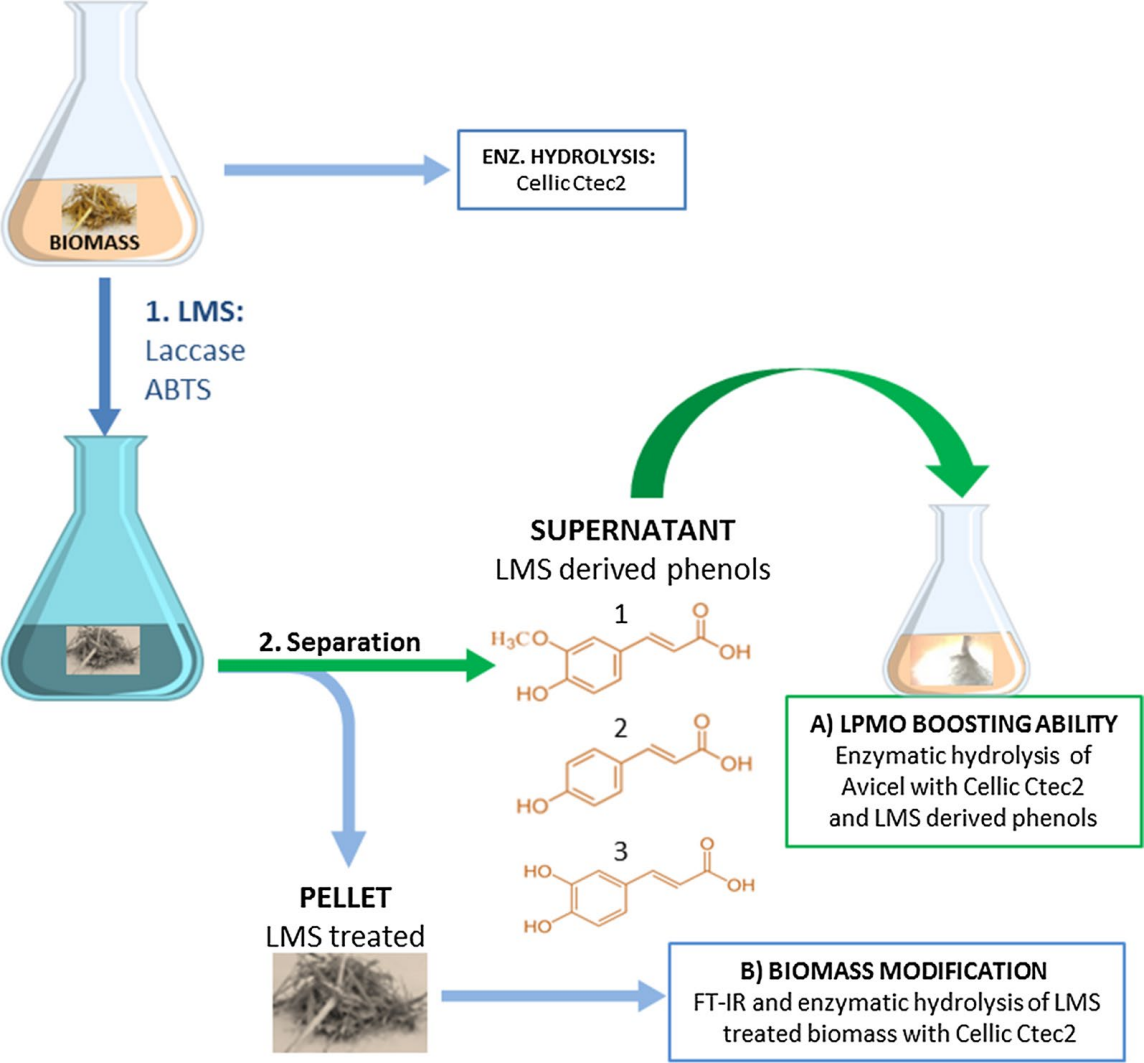
Flow chart of experiments investigating the ability of LMWLDC generated from laccase–mediator treatments on lignocellulose to boost LPMO activity and enhance commercial cocktail hydrolysis efficiency

In Fig. 1, the experimental concept is illustrated. The supernatants rich in LMWLDC as result of LMS treatment of SCB and WS biomass were added to the enzymatic hydrolysis of Avicel performed at 5% (w/v) DM in 0.05 M citrate buffer pH 5.5, in 1 mL reaction volume, at 50 °C, and 150 rpm using a rotary shaker. Cellic^®^ CTec2 was added at a dosage of 5 FPU g cellulose^−1^ DM, whereas 150 µL of each supernatant rich in LMWLDC from LMS or mediator-only treatment of SCB and WS biomass was added. Enzymatic hydrolysis of Avicel without LMWLDC-rich supernatants, or supplemented only with ascorbic acid (1 mM final concentration), was used as controls. After 72 h, the enzymatic hydrolysis was stopped by boiling the samples and analyzed with HPLC and HPAEC for quantification of glucose and its oxidized products.

The LMS-treated SCB and WS biomass separated and washed from their respective supernatants (pellets) were enzymatically hydrolyzed at 5% (w/v) DM in 0.05 M citrate buffer pH 5.5, in 1 mL reaction volume, at 50 °C, and 150 rpm using a rotary shaker. Cellic^®^ CTec2 was added to a dosage of 10 FPU g cellulose^−1^ DM. After 72 h, the enzymatic hydrolysis was stopped by boiling the samples and analyzed with analytical methods for quantification of monosaccharides and their oxidized products. All enzymatic hydrolyses were done in triplicate.

### Oxygen consumption measurements

Oxygen consumption measurements were performed using a Chlorolab 2 System (Oxy-Lab Hansatech^®^, England), with an oxygen sensor mounted at the bottom of a sealed reaction chamber and the agitation provided by a magnetic stirrer. The initial oxygen concentration (*T*_0min_) of each measurement was set as 100% and the following O_2_ consumption calculated relative to this amount. The measurements were performed by reproducing the same conditions applied for laccases treatments (paragraph 2.3) in terms of enzymes and ABTS dosages. WS substrate was loaded at 5% DM, 25 °C (± 0.5) was chosen as temperature to maximize the oxygen availability, and 100 rpm for the agitation and the reactor was kept sealed without oxygen supply. For the incubation of WS with Cellic^®^ CTec2, the same physical parameters (agitation temperature and solids content) were kept as for laccases, and the cocktail was added to a dosage of 10 FPU g cellulose^−1^ DM in 0.05 M citrate buffer pH 5.5. The control experiment was set containing WS sodium acetate buffer and ABTS (like in the LMS treatment) without enzymes.

### LMS and cellulases co-incubation experiments

#### Simultaneous LMS and enzymatic hydrolysis

SCB or WS was loaded at 5% (w/v) dry matter (DM) in 0.05 M sodium acetate buffer pH 5.0 in 1 mL reaction volume using 2 mL screw cup tubes at 50 °C temperature and 800 rpm shaking for 72 h. MtL or TvL laccases were added at a dosage of 10 U g cellulose^−1^ DM (corresponding to 500 µU mL^−1^ of enzyme) and the ABTS mediators were dosed at a final concentration of 1 mM. The cellulolytic cocktail Cellic^®^ CTec2 was added to a dosage of 10 FPU g cellulose^−1^ DM.

#### Separated LMS and enzymatic hydrolysis

WS was loaded at 5% (w/v) dry matter (DM) in 0.05 M sodium acetate buffer pH 5.0, in 1 mL reaction volume using 2 mL screw cup tubes at 50 °C temperature, and 800 rpm shaking together with MtL or TvL laccase added at a dosage of 10 U g cellulose^−1^ DM (corresponding to 500 µU mL^−1^ of enzyme) and the ABTS mediators was dosed at a final concentration of 1 mM. After 1 h, the laccase activity was stopped by adding NaN_3_ at a final concentration of 0.05% (w/v), and the reaction medium re-oxygenated by fluxing oxygen gas in the empty space of the reaction tube. Then, the cellulolytic cocktail Cellic^®^ CTec2 was added to a dosage of 10 FPU g cellulose^−1^ DM and the enzymatic hydrolysis was kept for 72 h.

#### Analytical methods

The glucose quantification was done using an Ultimate 3000 HPLC (Dionex, Germering, Germany) equipped with a refractive index detector (Shodex, Japan). The separation was performed with a Phenomenex Rezex ROA column, kept at 80 °C, with 5 mM H_2_SO_4_ as the mobile phase at a flow rate of 0.6 mL min^−1^. The results were analyzed using the software Chromeleon (Dionex).

The oxidized monosaccharide quantification was conducted with HPAEC chromatography using an ICS5000 system (Dionex, Sunnivale, CA, USA) equipped with a gold electrode PAD to analyze the oxidized products. The separation was performed with a CarboPac PA1 2 × 250 mm analytical column (Dionex, Sunnivale, CA, USA) and a CarboPac PAC1 2 × 50 mm guard column, maintained at 30 °C. The gradient mixing of eluents 0.1 M NaOH and 1 M NaOAc (sodium acetate) used has been described in detail previously [8, 14].

The GPC measured the molecular-weight distribution of the LMWLDC present in the supernatant. 200 µL of each supernatant was diluted in 200 µL in a 9:1 DMSO: water mixture containing 0.05 M LiBr (the mobile phase) and transferred to a sample vial. The GPC gradient was performed isocratically with a Hitachi 7000 system setup with a PolarSil column (300 mm, 5 μm particles), at 1 mL min^−1^ flow and 40 °C. Detection was obtained using a UV detector (280 nm), using tannic acid and phenol as external standards.

The LMWLDC-rich supernatants from LMS treatments of SCB and WS were also analyzed by Reverse Phase Liquid Chromatography (RP-HPLC). RP-HPLC was carried out with a Ascentis^®^ Express C18 column (15 cm × 2.1 mm, 2.7 μm) (Supelco™ Analytical, Bellefonte, PA, USA) on an Shimadzu HPLC system equipped with diode array detection at a flow rate of 0.5 mL min^−1^ and 40 °C. A gradient of buffer A (2% acetonitrile and 0.2% formic acid in water w/w) and buffer B (2% water and 0.2% formic acid in acetonitrile w/w) was used as follows: 0% B over 4 min, 10% B for 15 min, 50% B for 1 min, and 0% B over 20–50 min. The injection volume was 1 μL for samples and phenolic compounds (quinones, para-coumaric, caffeic and ferulic acid, vanillin, vanillic acid, and tannic acid) from Sigma-Aldrich^®^ were used as internal standards.

#### FT-IR and UV spectroscopy

The lignocellulosic materials recovered after LMS treatments were analyzed using a Thermo Nicolet 6700 FT-IR spectrophotometer equipped with a Golden Gate (diamond) ATR accessory and a DTGS (KBr) detector. Spectra were collected at room temperature in the 4000–800 cm^−1^ range with an average of 150 scans. A background of 150 scans was acquired, and the spectrum of each sample is reported as the average of three spectra of three biological replicates. FT-IR data were normalized by discounting the average between 1750 and 1800 cm^−1^, where there is no signal, and dividing by the maximum intensity value in the region from 1000 to 1250 cm^−1^. The lignin-derived compounds and phenolics present in the supernatant from LMS or mediator-only treatment on WS and SCB were also analyzed by UV in alkaline solution (pH > 12) to assure that the hydroxyl groups were ionized and the absorption changed towards longer wavelengths and higher intensities. The liquid fractions were diluted 36 times in NaOH 0.1 M and placed in a 1 cm quartz cuvette. The UV absorption spectrum (220–400 nm) was then recorded using an OceanView spectrophotometer UV–Vis (Ocean Optics^®^, The Netherlands). Reference solution was consistent with NaOH solution used for the samples.

## Results

### Laccase-mediated system (LMS) for isolation of low-molecular-weight lignin-derived compounds (LMWLDC)

Two laccases (MtL and TvL) together with a mediator molecule ABTS were used to generate LMWLDC from SCB and WS (as illustrated in Fig. 1). The supernatant rich in lignin-derived compounds was collected and further characterized [using UV-light absorbance, gel permeation chromatography (GPC), and reverse phase chromatography RP-HPLC] before using these as booster for LPMO during enzymatic hydrolysis of Avicel.

**Fig. 2:**
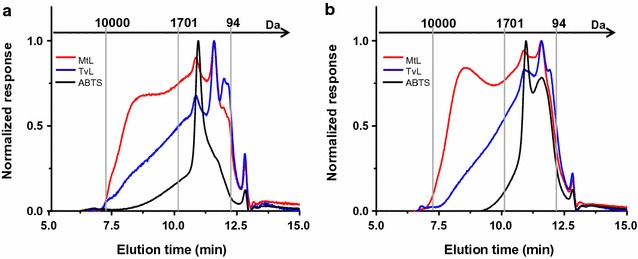
GPC chromatograms showing the molecular-weight distribution of the LMWLDC in the supernatants from LMS-treated SCB (**a**) and WS (**b**). Red lines are the incubations of the biomass with LMS based on MtL, and the blue lines are based on TvL laccases. Black lines refer to control experiments missing laccases. On top of the chromatograms are indicated the molecular-weight distributions in Dalton (Da) which are inversely correlated with the elution time: higher molecular weight corresponds to lower elution time. The 1701 and 94 Da are calculated measuring the elution time of tannic acid (10.5 min) and phenol (12.3 min), respectively

The increases in UV-light absorbance at 280 nm were detected in the supernatants of incubations of SCB or WS biomass with LMS compared to their controls lacking of laccases enzymes (results in Table 1). MtL laccases promoted an increase of more than 100% in UV absorbance from the initial values regardless the type of biomass. In addition, TvL laccases were active on both SCB and WS lignocelluloses, and the UV absorbance of the reaction medium increased 20% with the respect of their control reactions lacking of laccases. This confirmed that lignin was partially degraded, leading to release of smaller fragments into the supernatant of the reaction made of water as a solvent. The weight distributions of LMWLDC from the same sample are analyzed with GPC and are shown in Fig. 2. The spontaneous release of lignin-derived compounds from both biomasses lacking of LMS treatment (negative control) had a narrow molecular-weight distribution, with predominance of compounds with molecular weight ranging from 1000 to 1700 Da. The incubation with LMS increased the overall amount of compounds with a broad molecular-weight distribution between 100 and 10,000 Da.

**Table 1:** UV-Absorbances at 280 nm of liquid fractions of LMS-treated SCB or WS

Sample	Abs_280 nm_
Control SCB	0.67
SCB + LMS_MtL	1.39
SCB + LMS_TvL	0.80
Control WS	0.97
WS + LMS_MtL	1.89
WS + LMS_TvL	1.18

The LMS treatments of biomass are based on MtL or TvL laccases incubated with ABTS for 6 h. The control experiments lack of laccases but contained ABTS

RP-HPLC was used for the characterization and identification of lignin-derived monophenolic compounds in the supernatant of the reaction containing lignocellulose and LMS, and to confirm that all ABTS added was present at the end (Additional file 1: Figs. S1, S2; Tables S2, S3). Mainly, ferulic and coumaric acids were detected in the supernatants of WS and SCB when no LMS was present in the reaction, in agreement with the previous results [14, 29, 30]. However, after the incubation with LMS, these monophenolic compounds mostly disappeared, while traces of caffeic acid (for MtL), a good electron donor for LPMO [18], and few other unknown monophenols appeared. The peak corresponding to the ABTS in the chromatograms disappeared in the supernatants after LMS treatment (Additional file 1: Figs. S1, S2), which means that all the amounts of the mediator added at the beginning of the incubation were completely oxidized after 6 h. These results indicate that a depolymerization of insoluble lignin occurred, but also it cannot be ruled out that a re-polymerization phenomenon of the released monophenolics occurred at the same time, thus generating lignin-derived compounds with increased molecular weight (in agreement with a series of the previous papers [31–34]). This also explains the formation of an intermediate size of phenolic compounds around 600–1000 Da detected by the GPC but not detectable by the RP-HPLC tuned for smaller sized compounds. This pool of phenolics was suggested to be the responsible for the long-range electron transfer from lignin to LPMO enzymes [18].

### Low-molecular-weight lignin-derived compounds generated by laccase mediator system boost LPMO enzymes

**Fig. 3:**
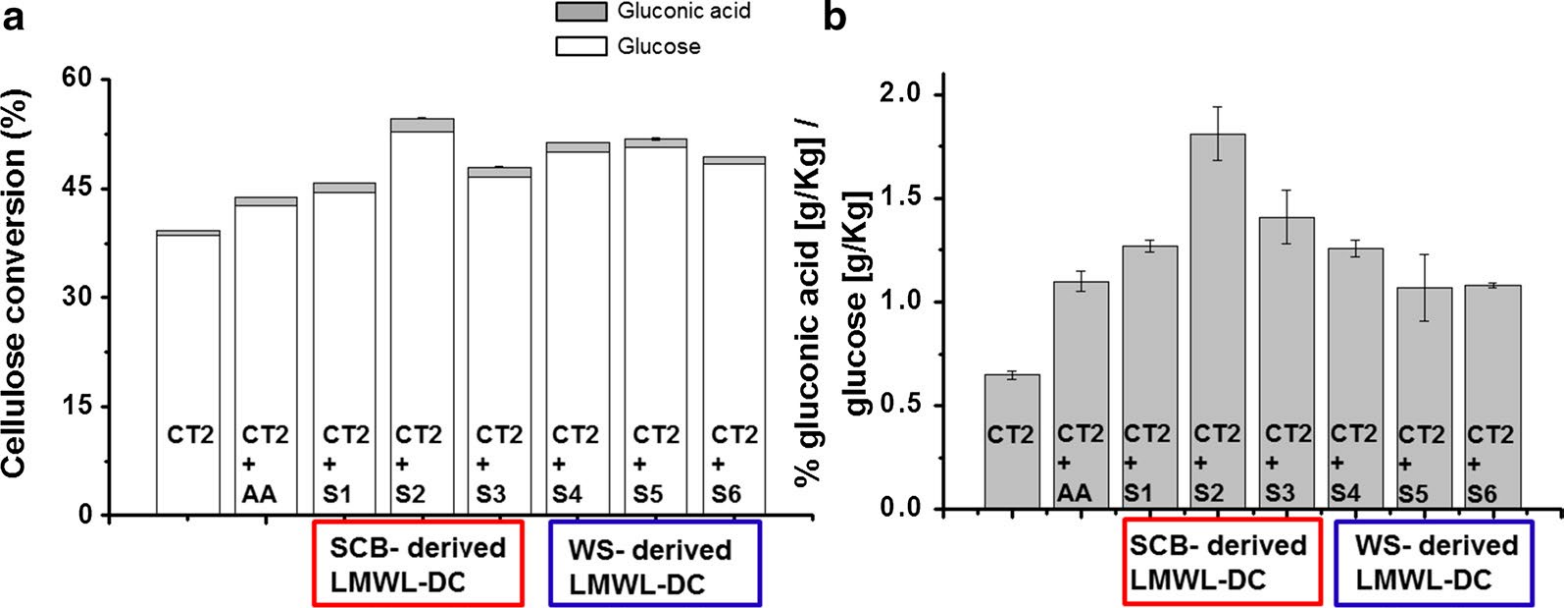
Enzymatic hydrolysis of Avicel incubated with supernatants rich in LMWLDC after LMS treatments of biomass at different conditions. The enzymatic hydrolysis was run with the LPMO containing cocktail Cellic^®^ CTec2 (CT2). **a** In the *y*-axis are reported the cellulose (white bars) and glucose oxidation (gluconic acid, grey bars) conversion yield in percentage of the maximum theoretical cellulose conversion after 72 h at 50 °C. **b** The *y*-axis shows the percentage of the amount of gluconic acid over the amount of glucose hydrolyzed from cellulose both quantified as g/Kg. LPMOs oxidize the cellulose resulting in a non-reducing end and a C1-oxidized end. The subsequent actions of exo-cellulases and β-glucosidases are glucose and gluconic acid (monomer of C1 oxidation). Thus, the *y*-axis represents the percentage of the cellulose oxidized over the total amount cellulose hydrolyzed. Error bars represent the standard errors based on the means of triplicate experiments. CT2: control experiment with Avicel and CT2 enzymes only; CT2 + AA: Avicel, CT2 enzymes and ascorbic acid (AA); S (supernatants): CT2 + S1: Avicel, CT2 enzymes and supernatant containing LMWLDC from incubation of SCB and ABTS; CT2 + S2: Avicel, CT2 enzymes and LMWLDC from incubation of SCB and LMS-MtL; CT2 + S3: Avicel, CT2 enzymes and LMWLDC from incubation of SCB and LMS-TvL; CT2 + S4: Avicel, CT2 enzymes and LMWLDC from incubation of WS and ABTS; CT2 + S5: Avicel, CT2 enzymes and LMWLDC from incubation of WS and LMS-MtL; CT2 + S6: Avicel, CT2 enzymes and LMWLDC from incubation of WS and LMS-TvL

The released LMWLDC, produced after LMS treatment on pre-treated SCB and WS, was isolated from the insoluble lignocellulose and added to the enzymatic hydrolysis of Avicel cellulose (experimental scheme in Fig. 1). The LPMO containing cocktail Cellic^®^ Ctec2 was used for the enzymatic hydrolysis, and its cellulose oxidizing activity was monitored detecting gluconic acid production (C1 oxidation of glucose). After 72 h of hydrolysis, it was observed that all the reactions incubated with supernatants derived from LMS and rich in LMWLDC, improved cellulose conversion correlating with an increase in LPMO activity (Fig. 3 and Additional file 1: Table S1) compared to the control experiments without any electron donors added. Notably, the cellulose conversion increased 42% for the Avicel hydrolysis when Cellic^®^ CTec2 was incubated with the LMWLDC released from pre-treated SCB incubated with MtL laccase (in comparison with the control lacking of LMWLDC) (Fig. 3). The Avicel hydrolysis yield increased 25% when using TvL laccase on SCB to generate LMWLDC. In addition, a positive response was obtained using LMWLDC from WS; the cellulose conversion increased 34% with the LMWLDC isolated with MtL laccase and also using the phenolics spontaneously released, while 29% increase was obtained with LMWLDC with TvL (Additional file 1: Table S1). Controls containing only ABTS at 0.15 mM (the same concentration present in the reactions incubated with LMWLDC-rich supernatants) were found effectless to the enzymatic hydrolysis and to the LPMO in agreement as previously reported [18] (data not shown).

**Fig. 4:**
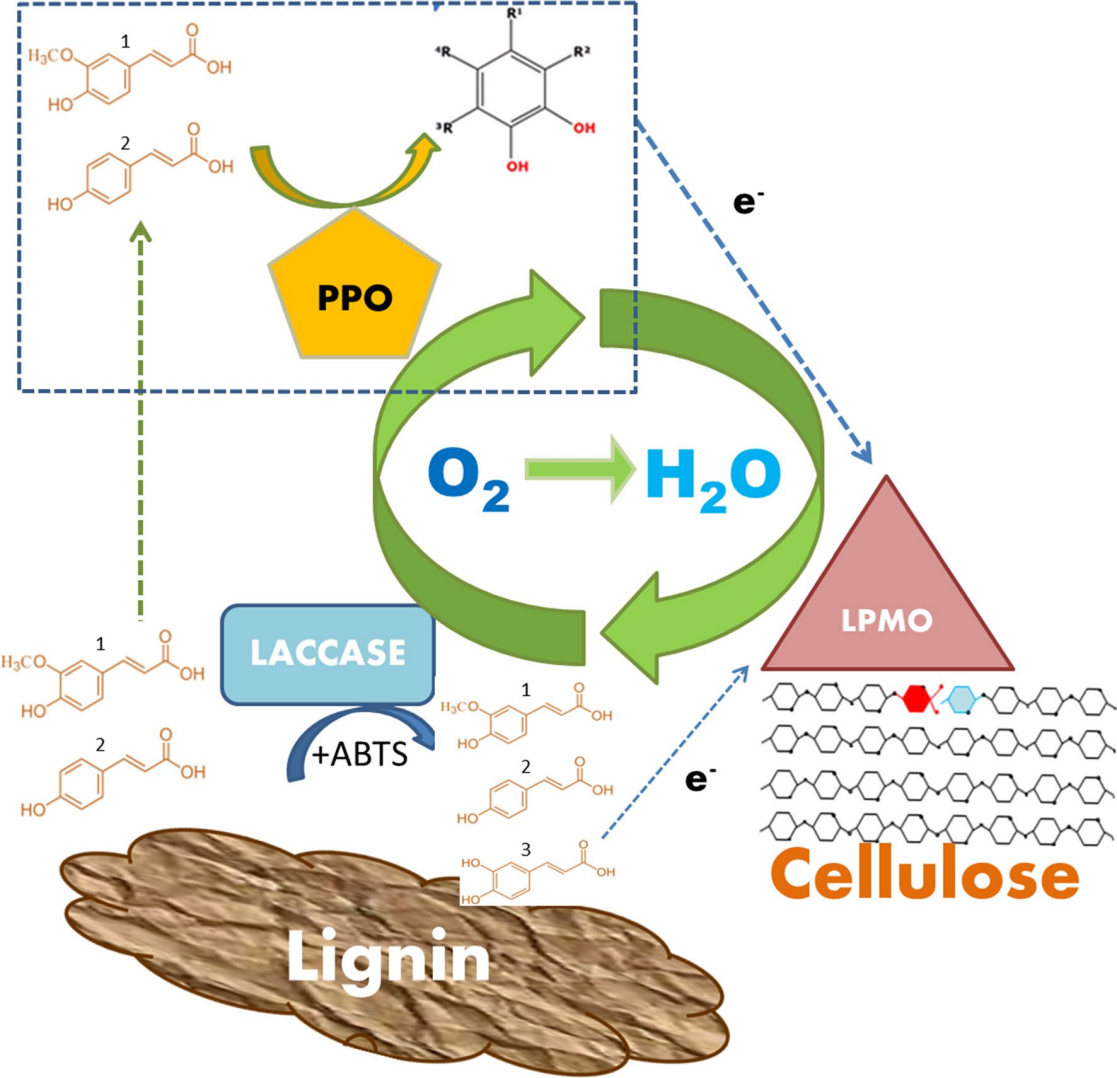
Simple representation of several oxidases enzymes degrading lignocellulose. The LMS system is depicted as Laccases + ABTS while depolymerizing the bulk lignin for the production of low-molecular-weight lignin-derived compounds (LMWLDC) represented by the compounds identified in this study: the (2) coumaric, (3) caffeic, and (1) ferulic acid, respectively, the hydroxylated and methoxylated form of coumaric acid. Since caffeic acid is a good electron donor to LPMO, it can directly activate the cellulose oxidation, while coumaric and ferulic acids do not have the same favorable redox potential. Thus, we hypothesize based on literature data that poly-phenol oxidase PPO can be used to hydroxylate the coumaric acid and other monophenols into good electron donor for LPMO, and at the same time mitigate the re-polymerization into high-molecular-weight lignin-derived compounds. All the enzymes depicted in the figure are all oxygen dependent (figure adapted from [4, 17])

The detection of gluconic acid confirmed that the LMWLDC worked as electron donors activating the LPMOs in the cellulolytic cocktail Cellic^®^ CTec2. The highest glucose oxidation was obtained for the supernatants containing the LMWLDC isolated from SCB and MtL (1.8%), followed by SCB and TvL (1.4%). Notably, these data correlate with the amount of released LMWLDC detected with UV_280_ absorbance (Table 1): at higher amount of LMWLDC corresponded the highest LPMO activity (detected as cellulose oxidation Additional file 1: Table S1, supernatant 2) and consequently a higher enzymatic hydrolysis of Avicel. In addition, the results correlate with the presence of caffeic acid in the supernatants, produced by the activity of MtL on SCB (detected with RP-HPLC) and not present before the LMS treatment (Additional file 1: Fig. S2; Table S3). Caffeic acid and in general plant-derived methoxylated and non-methoxylated compounds were previously found to be efficient LPMO-reducing agents [18, 34]. A recent paper [4] showed how poly-phenol oxidase (PPO) can boost LPMO activity by hydroxylating plant-derived monophenolics turning some phenolics and methoxylated phenolics into active reducing agent donating electrons to LPMO. Thus, in Fig. 4, we drew a simple model to implement the PPO activity in the wider scenario of lignocellulose oxidation/hydrolysis together with laccase and LPMO. Bulk insoluble lignin is the primary source of poly-phenols which are depolymerized by the LMS activity at the expense of oxygen, and the produced LMWLDC pool contains an heterogeneous distribution of phenolics (mono to trimers, and also methoxylated [18]) some of which can directly activate LPMO. But also some of the LMWLDC, if inefficient in donating electrons to LPMO, could represent a substrate for the PPO oxidase, which after an hydroxylation at the expense of oxygen, can turn these LMWLDC in good electron donors for LPMO [4]. Since also the cellulose oxidizing activity of LPMO is heavily dependent on oxygen, the amount of dissolved oxygen and or anaerobic versus aerobic conditions should be carefully considered for any application or co-incubation of these oxidases together. Thus, the next paragraph is dedicated to applications of laccase and LPMO co-incubations focusing on their competition over oxygen.

### Co-incubation of laccase and LPMO: oxygen competition

**Fig. 5:**
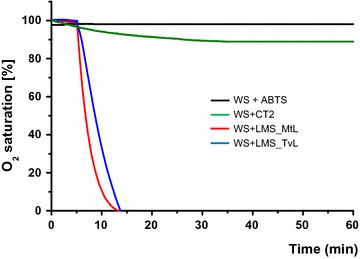
Oxygen consumption measurements as percentage decrease of O_2_ saturated solution versus time. Each line is representative of average of triplicate experiments containing: WS and ABTS (black); WS and Cellic^®^ CTec2 (green); and WS, LMS_MtL laccase (red), WS, LMS_TvL laccase (blue)

When co-incubating laccases and LPMOs on lignocellulosic substrates, their O_2_ consumption rates should be considered to avoid any competition. Enzymatic reactions were set in a 1 ml sealed reactor chamber equipped with an oxygen electrode disc as sensor to monitor the oxygen consumption. In this reactor, it was possible to perform simultaneous hydrolysis and oxidation of lignocellulosic substrate, i.e., WS or the pure cellulose Avicel. As shown in Fig. 5, 50% of the initial oxygen was consumed in 6.8 min during the incubation of WS with MtL laccase and 8.7 min for TvL laccase both at 25 °C of temperature. In 15 min, both laccases consumed all the dissolved oxygen to undetectable levels. In comparison for the LPMOs, only 10% of the oxygen available was consumed during the first 26 min when WS was incubated with Cellic^®^CTec2.

**Fig. 6:**
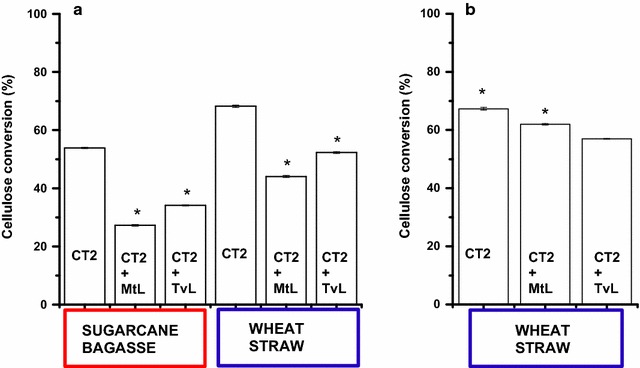
Enzymatic hydrolysis yields. **a** Simultaneous LMS and enzymatic hydrolysis of lignocellulose: samples containing pre-treated sugarcane bagasse and Cellic^®^ CTec2 (CT2); CT2 + MtL; and CT2 + TvL are SCB treated with LMS based on either MtL or TvL laccases, respectively. The same legend has been used for pre-treated wheat strawhydrolyzed with Cellic^®^ CTec2 (CT2), while CT2 + MtL and CT2 + TvL indicate the addition of LMS based on MtL and TvL, respectively. **b** Separated LMS (first), re-oxygenation, and enzymatic hydrolysis (after) of wheat straw. CT2 was hydrolyzed with Cellic^®^ CTec2 without any LMS treatment; while CT2 + MtL and CT2 + TvL indicate pre-incubation with LMS based on MtL and TvL, respectively. Then, after inactivation of laccases with sodium azide, wheat straw was hydrolyzed with Cellic^®^ CTec2. (*) The mean difference is statistically significant at the 0.05 level by the Tukey test

Based on these results, we could predict an inhibition of the LPMO enzymes contained in the cellulolytic cocktails during the hydrolysis of WS and SCB lignocellulosic materials if co-incubated with laccases. The same setup used in the previous experiment to generate LMWLDC with LMS was repeated on WS and SCB and simultaneously Cellic^®^ CTec2 was added. In fact, a decrease of enzymatic hydrolysis yield was observed: for SCB, 53.9% of conversion yield was obtained for control experiment incubated solely with the cellulolytic cocktail, while when MtL laccases was added, only 27.3% of conversion was obtained, and 34.1% when incubated with TvL (Fig. 6a). When using hydrothermally pre-treated WS as substrate, the inhibition of the enzymatic hydrolysis was similar: 68.1% of conversion was obtained for the control hydrolysis with the cellulolytic cocktail alone, whereas 44.1 and 52.3% were obtained when adding MtL and TvL, respectively (Fig. 6a). The LPMOs were inhibited by the anoxic environment despite the presence of electron donor molecules like LMWLDC produced by the laccase. Noteworthy is the correlation between the activity of the laccases generating LMWLDC reported in Table 1 (despite the biomass employed) and the inhibition of the enzymatic hydrolysis caused. MtL laccases were found to be more active on SCB, which reported the lowest cellulose enzymatic conversion.

To avoid the oxygen competition between laccase and LPMO, the two enzymatic reactions were separated, but performed on the same material. The hydrothermal pre-treated WS was incubated with LMS, and after the inhibition of the laccase by adding sodium azide harmless to cellulolytic cocktail [35] and to LPMO (data not shown), the reaction medium was re-oxygenated, and the LPMO containing cellulolytic cocktail was added. Results similar to the control experiment were obtained when using this separated incubation: MtL laccase-based LMS led at 62% of cellulose hydrolysis yield compared to 68% when using Cellic^®^ CTec2 only, restoring 74% of the cellulose hydrolysis yield lost with the simultaneous co-incubation strategy (Fig. 6b). When TvL laccase was used in LMS, the final glucose yield was 57% in the separated approach. Despite an active LPMO, i.e., production of gluconic acid in the presence of LMWLDC electron donors and oxygen, the enzymatic hydrolysis yield did not increased when comparing LMS-aided towards standard enzymatic hydrolysis (Cellic^®^ CTec2 only). This indicates that laccase can be applied for the production of electron donors from lignin to boost LPMO, but it might also cause other inhibiting effects, e.g., a modification of cellulose fibers which could render the lignocellulose more recalcitrant to enzymatic hydrolysis as previously observed [29–31, 36], or by increasing the binding of cellulases on modified lignin [37, 38].

### Insights on LMS detrimental effect on cellulose conversion

The negative results obtained with the co-incubation of LMS and Cellic^®^ CTec2 over WS and SCB material, and partially restored with a separation of the laccase incubation and LPMO containing cocktail, made us wonder if LMS could modify cellulose fibers contributing to the inhibition of the enzymatic hydrolysis. Pure cellulose (Avicel) was treated with LMS using both MtL and TvL laccases. The resulting cellulose was hydrolyzed with Cellic^®^ CTec2 and the hydrolysis yield was found to be lower: 57 and 52% for MtL- and TvL-treated Avicel (respectively) compared to Avicel without LMS treatment (66%) (Additional file 1: Fig. S3a). Prior to the enzymatic hydrolysis, the LMS-treated Avicel was thoroughly washed and the surface analyzed with FT-IR spectroscopy. FT-IR analysis (Additional file 1: Fig. S3) revealed that LMS treatment of Avicel cellulose based on TvL laccase decreased by 32% the hydroxyl stretching band at 3335 cm^−1^ and by 25% the band at 2850 cm^−1^ related to the symmetrical stretching vibrations of CH_2_ groups (Additional file 1: Table S4). No difference was observed for LMS treatment of Avicel by MtL laccase compared to the untreated Avicel. These modifications suggest that LMS treatment with TvL laccase may have decreased the free hydroxyl groups on the cellulose surface, or inter-fiber covalent bonds through hemiacetal linkages between hydroxyl groups and carbonyl groups could be formed increasing the strength of the cellulose [37, 39–41]. RP-HPLC analysis of the liquid phase also showed that all ABTS added at the beginning of the reaction was present in the supernatant and at the end of the LMS incubation period (results not shown) and nor oxidized or grafted onto the material.

It has already been shown that oxidized groups on cellulose such as carbonyl and carboxyl groups can decrease the enzymatic hydrolysis of cellulose, inhibiting cellobiohydrolases and β-glucosidases [31]. Here, we report that the modification of OH and CH_2_ groups on the surface of Avicel caused by the LMS with a high redox potential laccase can also negatively affect the enzymatic hydrolysis yield. FT-IR data of SCB and WS after LMS treatment with TvL laccase also revealed a decrease in the hydroxyl content by 17 and 50% for SCB and WS (Additional file 1: Figure S5a, b, respectively, and Additional file 1: Table S4) in cellulose surface. In agreement with the results observed for Avicel, the hydrolysis yield was found to be lower (15%) for TvL-treated WS compared to the untreated material (Additional file 1: Fig. S4). As observed in the previous studies [37, 42–44], it seems that reducing hydroxyl groups in lignocellulose surface is a hallmark of LMS treatment.

Low redox potential laccases (i.e., MtL) should be preferred over high redox potential laccase (i.e., TvL) given the reduced extends of modification caused onto cellulose fibers. These modifications were found to be a key factor in increasing the recalcitrance of cellulose to the enzymatic hydrolysis. These results are in agreement with a recently published paper [44], where similar dosage of laccase caused negative enzymatic hydrolysis of cellulose when using enzymatic cocktail lacking of LPMO.

## Conclusions

The results obtained in this work show that the lignin-derived compounds and phenols released using laccase–mediator system were able to boost LPMO activity present in a commercial cellulolytic cocktail, increasing their hydrolysis efficiency of cellulose. In particular, the MtL laccase with low redox potential caused the highest release of low-molecular-weight lignin-derived compounds capable of activating LPMOs. It was also found that the co-incubation of laccase together with LPMOs containing cellulolytic cocktail led to substrate competition towards oxygen, causing an inhibition of LPMO. Thus, the LMS was applied prior to the cellulolytic enzymes on the same lignocellulosic material: the inhibition of the LPMOs was mitigated, but the overall cellulose hydrolysis did not increase. These results suggest the presence of a second inhibition caused by laccases but acting directly on the cellulose material. Laccase, especially with high redox potential TvL, could induce further chemical modification in lignocellulosic fibers increasing the recalcitrance of sugarcane bagasse and wheat straw. In conclusions for an affective exploitation of a long-range electron transfer from lignin to cellulose catalyzed by several oxidases, the enzymes should be chosen and dosed very carefully considering the substrate competition towards oxygen aiming at a co-incubation with LPMO. Moreover, the data obtained on lignin depolymerization after LMS activity suggest a potential synergy also with other oxidases (i.e., PPO) in producing low-molecular-weight lignin-derived compounds acting as a reducing agent for LPMO.

## Additional file

**Additional file 1: Table S1.** Enzymatic hydrolysis of Avicel supplemented with supernatants rich in low-molecular-weight lignin-derived compounds. Quantification of gluconic acid in g/Kg (first column), relative yield of cellulose oxidation calculated as % of the amount of gluconic acid in [g/kg]/ glucose [g/kg]. **Figure S1.** RP-HPLC analysis of the supernatants obtained for the pre-treated sugarcane bagasse incubated with buffer and ABTS (black line), LMS_MtL (blue line), or LMS_TvL (pink line): chromatograms relative to 308 nm wavelength. The new peaks that appeared after LMS treatment are circled by red circles. **Table S2.** List of compounds detected by RP-HPLC analysis from the supernatant obtained for the pre-treated sugarcane bagasse incubated with buffer and ABTS (Control), LMS_TvL, and LMS_MtL. The retention time, wavelength of maximum absorption and peak area, and peak height for each compound are reported. The height of each peak is in proportion to the amount of the component present in the sample mixture. **Figure S2.** RP-HPLC analysis of the supernatants obtained for the pre-treated wheat straw incubated with buffer and ABTS (black line), LMS_MtL (blue line), or LMS_TvL (pink line): chromatograms relative to 308 nm wavelength. The new peaks that appeared after LMS treatment are circled by red circles. **Table S3.** List of compounds detected by RP-HPLC analysis from the supernatant obtained for the pre-treated wheat straw incubated with buffer and ABTS (Control), LMS_TvL, and LMS_MtL. The retention time, wavelength of maximum absorption and peak area, and peak height for each compound are reported. The height of each peak is in proportion to the amount of the component present in the sample mixture. **Table S4.** Relative absorbance of bands in the infrared spectrum of different groups in the control experiment contained Avicel and ABTS, Avicel treated with LMS based on MtL laccase (Avicel + LMS_MtL) and LMS based on TvL laccase (Avicel + LMS_TvL). The data shown are from the normalized spectra. **Figure S3.** Enzymatic hydrolysis (A) and Fourier transform-infrared spectra (B**)** of Avicel treated with LMS based on MtL laccase (Avicel + LMS_MtL in A; red line in B) and LMS based on TvL laccase (Avicel + LMS_TvL in A, blue line in B), the control experiment contained Avicel and ABTS, lacking of laccases (Avicel in A; black line in B). (*) The mean difference is statistically significant at the 0.05 level by the Tukey test. The arrows indicate the main changes in the spectra after LMS treatment. **Figure S4.** Cellulose conversion % of hydrothermal sugarcane bagasse and wheat straw treated with laccase mediator system, followed by laccase inactivation, washing procedure and hydrolyzed using Cellic^®^ CTec2 for 72 h. Error bars represent the standard errors of the means of triplicate experiments. Legend: CT2—Cellic^®^ CTec2, SCB—pre-treated sugarcane bagasse, WS—pre-treated wheat straw, LMS_MtL—MtL laccase mediator system treatment, LMS_TvL—TvL laccase mediator system treatment, ABTS—only mediator treatment. **Figure S5.** Fourier transform-infrared (FT-IR) spectra of pre-treated sugarcane bagasse (A) and wheat straw (B) after ABTS only (black), LMS_MtL (red), and LMS_TvL (blue) treatment. **Table S5.** Relative absorbance of bands in the infrared spectrum of different groups in the untreated and LMS-pre-treated sugarcane bagasse and wheat straw samples. Legend: SCB: sugarcane bagasse, WS: wheat straw, LMS_MtL—MtL laccase mediator system treatment, LMS_TvL—TvL laccase mediator system treatment.*From the normalized spectra.
