# Supplementary material for: Laccase-derived lignin compounds boost cellulose oxidative enzymes AA9
Source: Biotechnol Biofuels. 2018 Jan 17;11:10. doi: 10.1186/s13068-017-0985-8 (PMC5771016; doi:10.1186/s13068-017-0985-8)
Supplement: Supplementary file 1 — Additional file 1: Table S1. Enzymatic hydrolysis of Avicel supplemented with supernatants rich in low-molecular-weight lignin-derived compounds. Quantification of gluconic acid in g/Kg (first column), relative yield of cellulose oxidation calculated as % of the amount of gluconic acid in [g/kg]/ glucose [g/kg]. Figure S1. RP-HPLC analysis of the supernatants obtained for the pre-treated sugarcane bagasse incubated with buffer and ABTS (black line), LMS_MtL (blue line), or LMS_TvL (pink line): chromatograms relative to 308 nm wavelength. The new peaks that appeared after LMS treatment are circled by red circles. Table S2. List of compounds detected by RP-HPLC analysis from the supernatant obtained for the pre-treated sugarcane bagasse incubated with buffer and ABTS (Control), LMS_TvL, and LMS_MtL. The retention time, wavelength of maximum absorption and peak area, and peak height for each compound are reported. The height of each peak is in proportion to the amount of the component present in the sample mixture. Figure S2. RP-HPLC analysis of the supernatants obtained for the pre-treated wheat straw incubated with buffer and ABTS (black line), LMS_MtL (blue line), or LMS_TvL (pink line): chromatograms relative to 308 nm wavelength. The new peaks that appeared after LMS treatment are circled by red circles. Table S3. List of compounds detected by RP-HPLC analysis from the supernatant obtained for the pre-treated wheat straw incubated with buffer and ABTS (Control), LMS_TvL, and LMS_MtL. The retention time, wavelength of maximum absorption and peak area, and peak height for each compound are reported. The height of each peak is in proportion to the amount of the component present in the sample mixture. Table S4. Relative absorbance of bands in the infrared spectrum of different groups in the control experiment contained Avicel and ABTS, Avicel treated with LMS based on MtL laccase (Avicel + LMS_MtL) and LMS based on TvL laccase (Avicel + LMS_TvL). The data shown are f [file 13068_2017_985_MOESM1_ESM.docx]

**SUPPLEMENTARY MATERIAL**

***Biotechnology for Biofuels***

**Laccase-derived lignin compounds boost cellulose oxidative enzymes AA9**

Lívia Brenelli^1,2,3^, Fabio M. Squina^3^, Claus Felby^1^ & David Cannella^1,2,4*^

1 University of Copenhagen, Faculty of Science, Department of Geosciences and Natural Resource Management, Frederiksberg C, Denmark.

2 Brazilian Bioethanol Science and Technology Laboratory (CTBE), Center for Research in Energy and Materials (CNPEM), Campinas, Brazil.

3 State University of Campinas, School of Chemical Engineering, Department of Process Engineering, Campinas, Brazil.

4 Present address: Interfaculty School of Bioengineering, Université Libre de Bruxelles (ULB), Campus Plaine CP242, Boulevard du Triomphe, Bruxelles 1050

*corresponding author: [david.cannella@ulb.ac.be](mailto:david.cannella@ulb.ac.be)

**Table S1.** Enzymatic hydrolysis of Avicel supplemented with supernatants rich in low molecular weight lignin-derived compounds. Quantification of gluconic acid in g/Kg (first column), relative yield of cellulose oxidation calculated as % of the amount of gluconic acid in [g/kg]/ glucose [g/kg].

| **Sample** | **Gluconic acid g Kg^-1^** | **Gluconic acid [g/Kg] /  glucose [g/Kg] *** | **Cellulose Conversion (%)** |
| --- | --- | --- | --- |
| CT2 | 0.14 ± 0.01 | 0.65 ± 0.02 | 37.3 ± 0.2 |
| CT2 + AA | 0.35 ± 0.01 | 1.10 ± 0.05 | 42.7 ± 0.8 |
| CT2 + supernatant 1 (SCB+ABTS no laccase) | 0.31 ± 0.01 | 1.27 ± 0.03 | 44.5 ± 0.4 |
| CT2 + supernatant 2 (SCB+LMS_MtL) | 0.53 ± 0.04 | 1.81 ± 0.13 | 52.9 ± 0.9 |
| CT2 + supernatant 3  (SCB+LMS_TvL) | 0.36 ± 0.03 | 1.41 ± 0.13 | 46.6 ± 0.1 |
| CT2 + supernatant 4  (WS+ABTS no laccase) | 0.35 ± 0.01 | 1.26 ± 0.04 | 50.0 ± 0.8 |
| CT2 + supernatant 5  (WS+LMS_MtL) | 0.30 ± 0.05 | 1.07 ± 0.16 | 50.7 ± 0.7 |
| CT2 + supernatant 6  (WS+LMS_TvL) | 0.29 ± 0.02 | 1.08 ± 0.01 | 48.3 ± 0.3 |
| *The glucose oxidation was calculated as percentage of the amount of gluconic acid over the amount of glucose hydrolyzed from cellulose. | | | |

**Figure S1.** RP-HPLC analysis of the supernatants obtained for the pre-treated sugarcane bagasse incubated with buffer and ABTS (black line), LMS_MtL (blue line) or LMS_TvL (pink line): chromatograms relative to 308 nm wavelength. The new peaks that appeared after LMS treatment are circled by red circles.


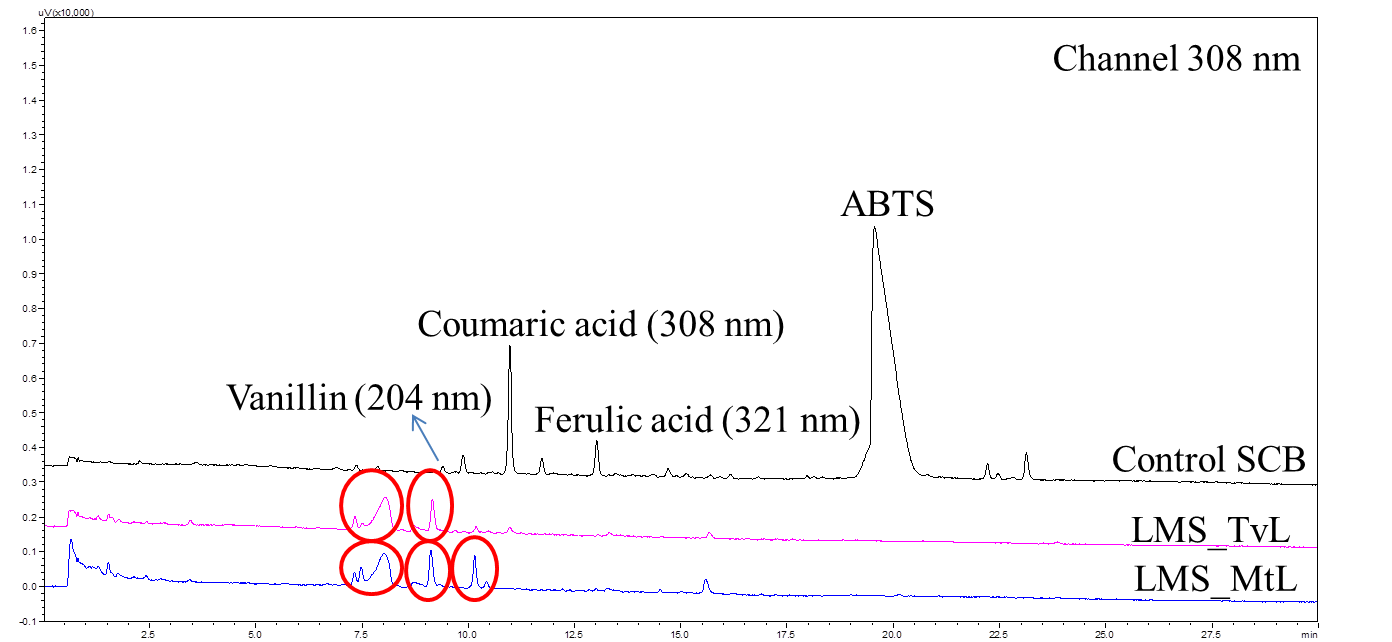


**Table S2.** The list of compounds detected by RP-HPLC analysis from the supernatant obtained for the pre-treated sugarcane bagasse incubated with buffer and ABTS (Control), LMS_TvL and LMS_MtL. The retention time, wavelength of maximum absorption and peak area and peak height for each compound is reported. The height of each peak is in proportion to the amount of the component present in the sample mixture.

**
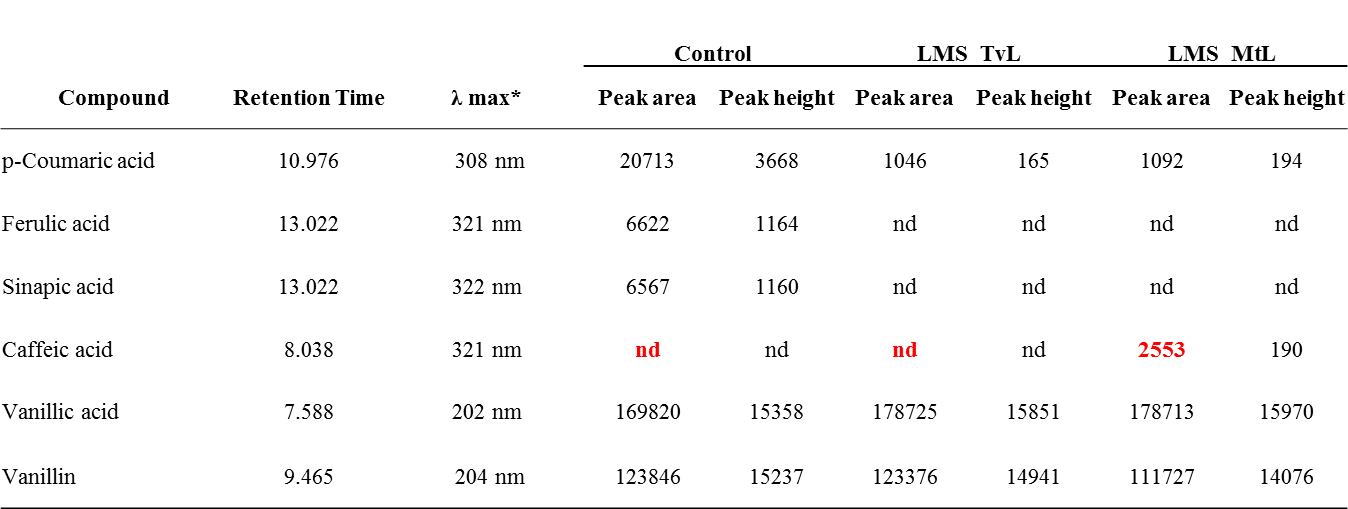
**

*wavelength of maximum absorbance; ND – not detected.

**Figure S2.** RP-HPLC analysis of the supernatants obtained for the pre-treated wheat straw incubated with buffer and ABTS (black line), LMS_MtL (blue line) or LMS_TvL (pink line): chromatograms relative to 308 nm wavelength. The new peaks that appeared after LMS treatment are circled by red circles.


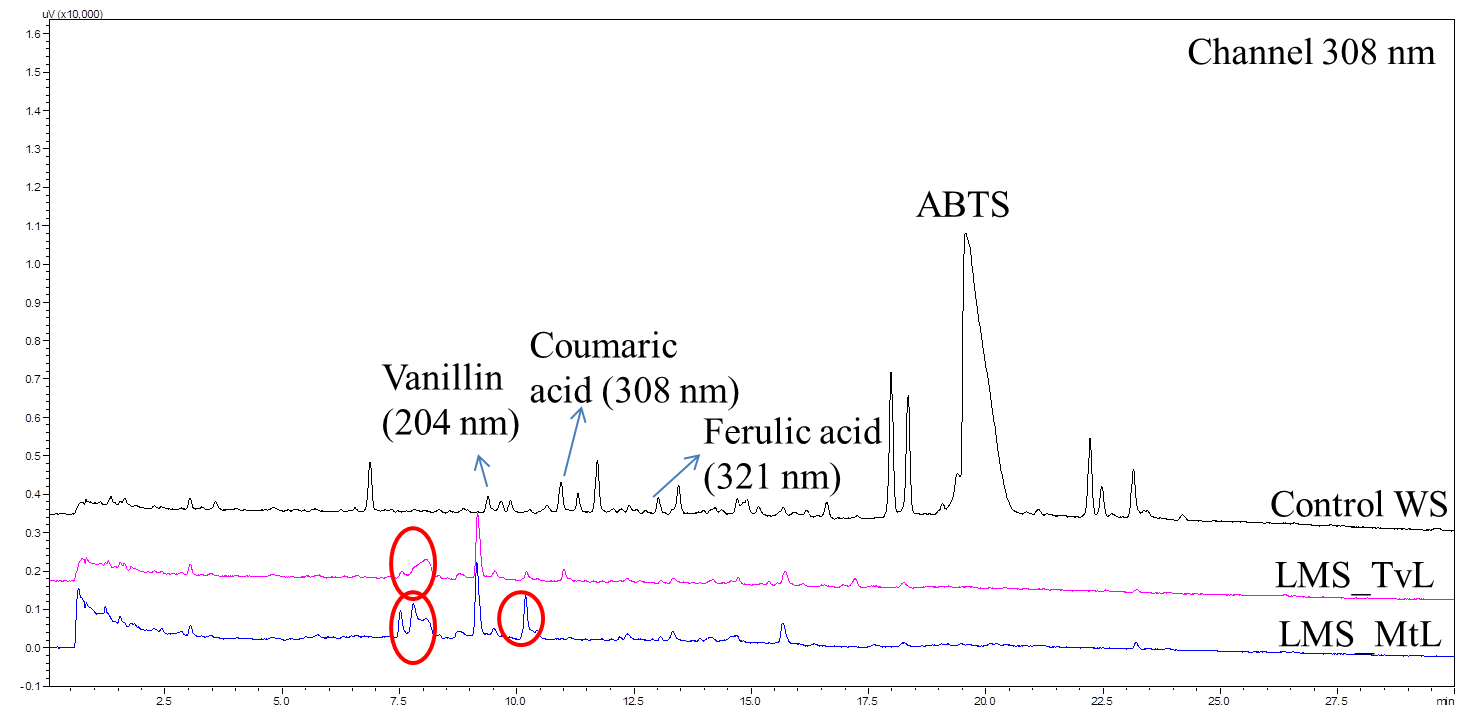


**Table S3.** The list of compounds detected by RP-HPLC analysis from the supernatant obtained for the pre-treated wheat straw incubated with buffer and ABTS (Control), LMS_TvL and LMS_MtL. The retention time, wavelength of maximum absorption and peak area and peak height for each compound is reported. The height of each peak is in proportion to the amount of the component present in the sample mixture.

**
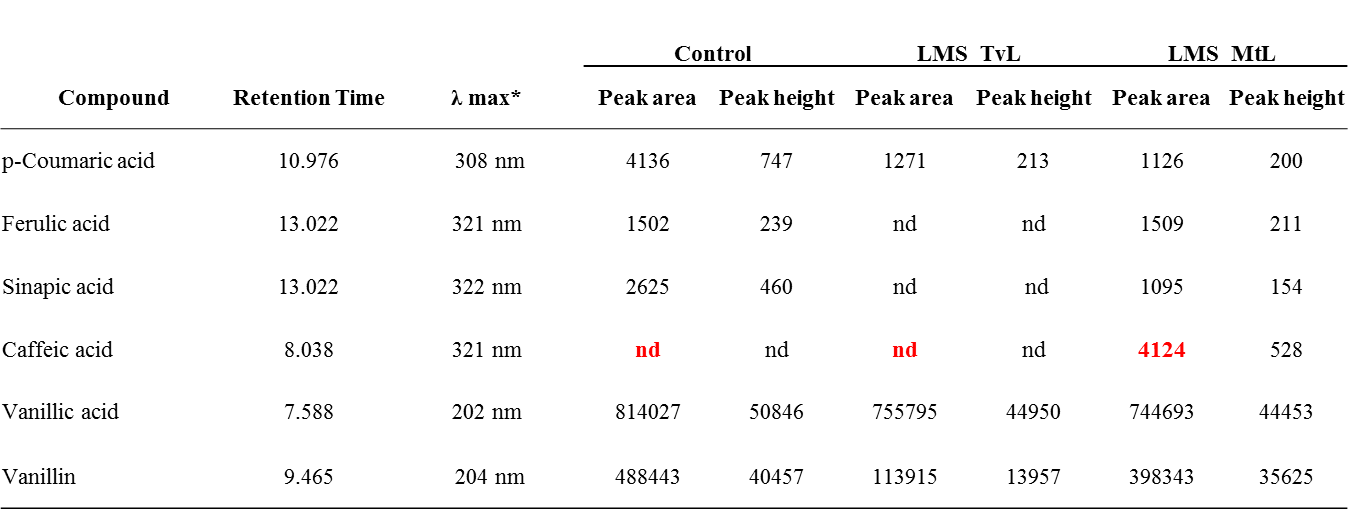
**

*wavelength of maximum absorbance; ND – not detected.

**Table S4.** Relative absorbance of bands in the infrared spectrum of different groups in the control experiment contained Avicel and ABTS, Avicel treated with LMS based on MtL laccase (Avicel+LMS_MtL) and LMS based on TvL laccase (Avicel+LMS_TvL). The data shown are from the normalized spectra.

| **Wavelenght** | **3335 cm^-1^** | **2850 cm^-1^** | **1105 cm^-1^** |
| --- | --- | --- | --- |
| Assignment | OH stretching | CH_2_  symmetrical stretching | C-O-C glycosidic |
| Relative Absorbance* | | | |
| Avicel + ABTS | 0.28 | 0.09 | 0.52 |
| Avicel + LMS_MtL | 0.26 | 0.08 | 0.56 |
| Avicel + LMS_TvL | 0.19 | 0.06 | 0.53 |

Legend: LMS_MtL – MtL laccase mediator system treatment, LMS_TvL – TvL laccase mediator system treatment. *From the normalized spectra.

**Figure S3.** Enzymatic hydrolysis (A) and Fourier transforms infrared spectra (B) of Avicel treated with LMS based on MtL laccase (Avicel + LMS_MtL in A; red line in B ) and LMS based on TvL laccase (Avicel + LMS_TvL in A, blue line in B), the control experiment contained Avicel and ABTS, lacking of laccases (Avicel in A; black line in B). (*) The mean difference is statistically significant at the 0.05 level by the Tukey test.The arrows indicate the main changes in the spectra after LMS treatment.

**
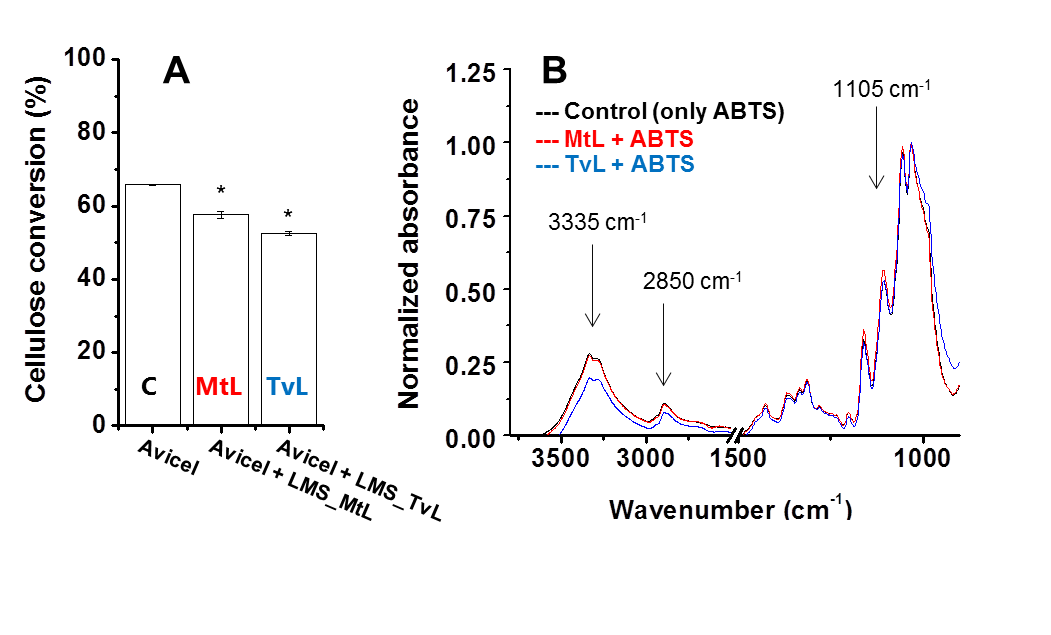
**

**Figure S4.** Cellulose conversion % of hydrothermal sugarcane bagasse and wheat straw treated with laccase mediator system, followed by laccase inactivation, washing procedure and hydrolyzed using Cellic^®^ CTec2 for 72h. Error bars represent the standard errors of the means of triplicate experiments. Legend: CT2 - Cellic^®^ CTec2, SCB – pre-treated sugarcane bagasse, WS – pre-treated wheat straw, LMS_MtL – MtL laccase mediator system treatment, LMS_TvL – TvL laccase mediator system treatment, ABTS – only mediator treatment.


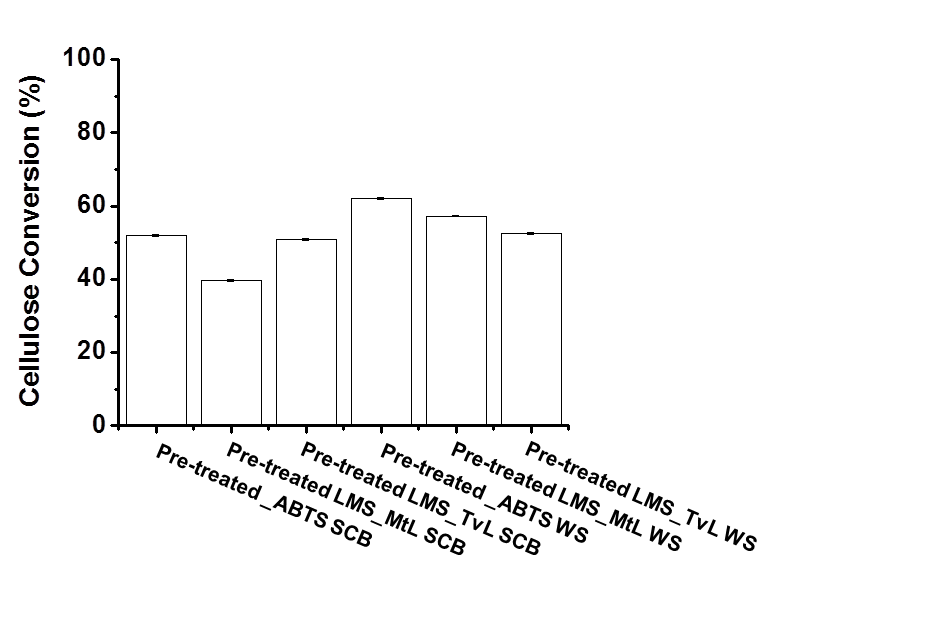


**Figure S5.** Fourier transforms infrared (FTIR) spectra of pre-treated sugarcane bagasse (A) and wheat straw (B) after ABTS only (black), LMS_MtL (red) and LMS_TvL (blue) treatment.


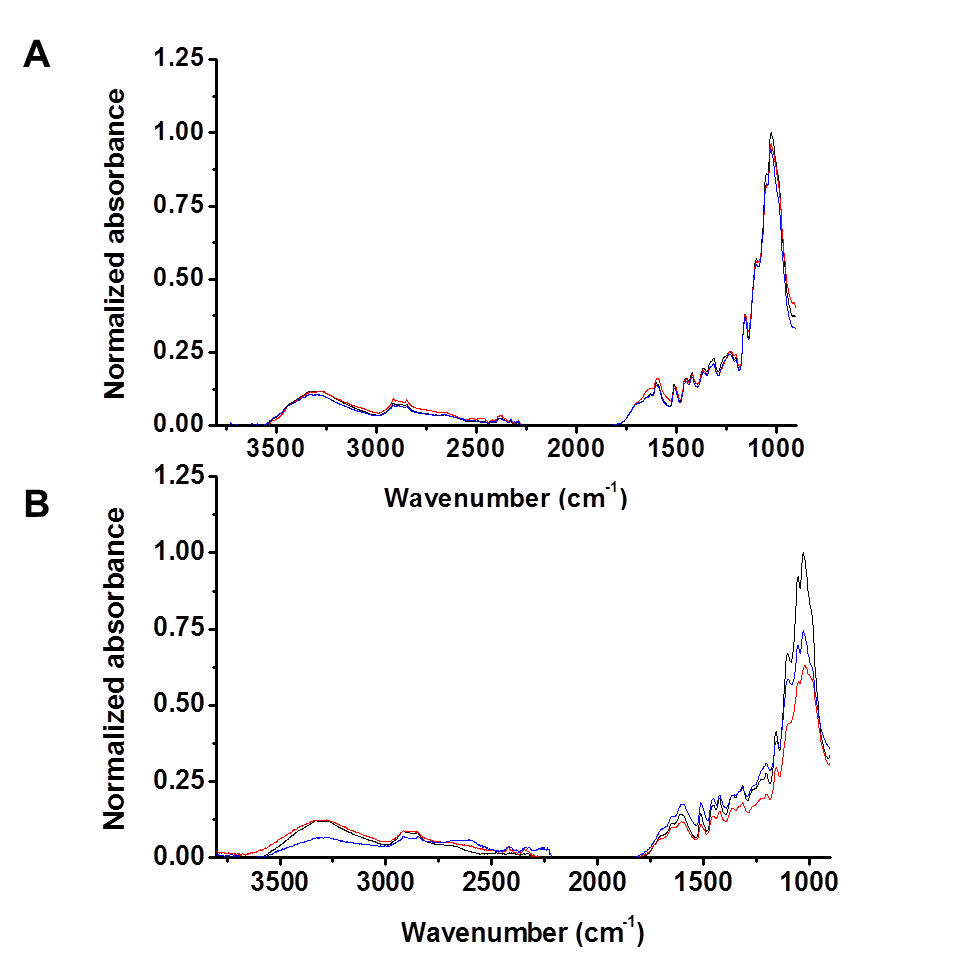


**Table S5.** Relative absorbance of bands in the infrared spectrum of different groups in the untreated and LMS -pretreated sugarcane bagasse and wheat straw samples. Legend: SCB: sugarcane bagasse, WS: wheat straw, LMS_MtL – MtL laccase mediator system treatment, LMS_TvL – TvL laccase mediator system treatment.*From the normalized spectra.


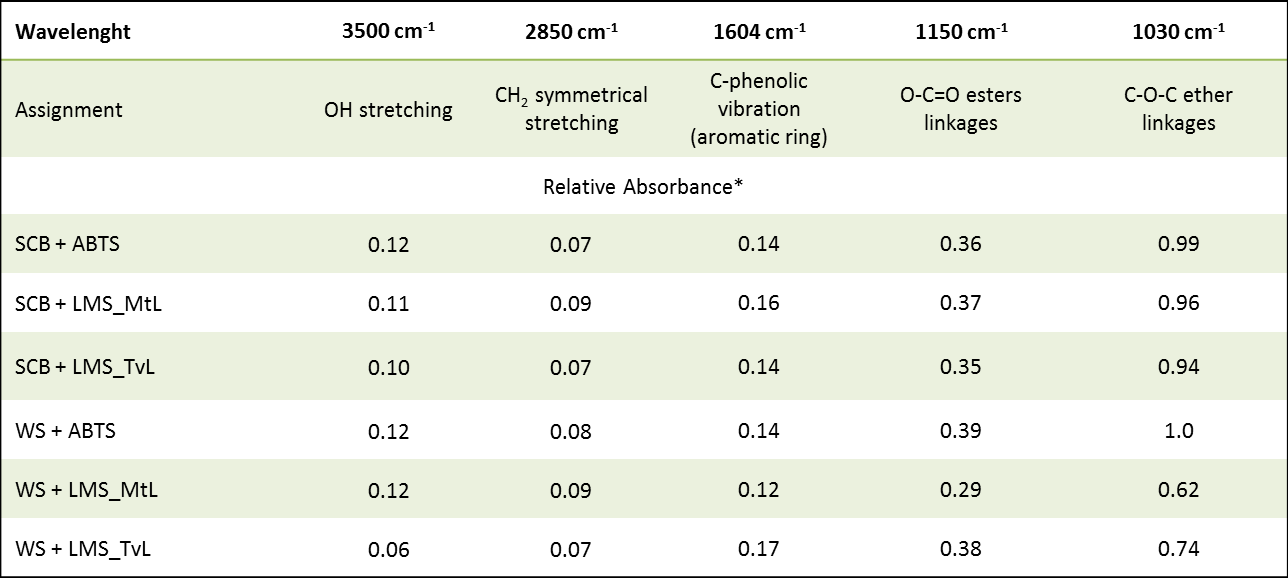


Legend: SCB: sugarcane bagasse, WS: wheat straw, LMS_MtL – MtL laccase mediator system treatment, LMS_TvL – TvL laccase mediator system treatment.*From the normalized spectra.
